# Loot

**Published:** 1868-02-01

**Authors:** 


### *LOOT.*

Dr. McGraw, Surgeon of Harper Hospital, calls attention (*Detroit Review*,) to the evils arising from attempts at removing, by cathartics, the constipation so frequently following severe surgical operations. He is in the habit, in such cases, of deferring all attempts to move the bowels until the cause of the constipation, the *excessive irritation of the nervous system*, has passed away. In cases where cathartics had been tried in vain he has succeeded in procuring passages by the administration of *Morphine* in large doses. In one case of the kind he reports securing free evacuations by hypodermic injection of half a grain of *Morphia*—the part selected being the skin of the abdomen. The constipations and diarrhœas of the army, he is convinced, were oftener the subjects of mal-practice on the part of men in good standing in the profession, than almost any other class of diseases. Sensible and true.

Dr. Chase (*St. Louis M. Rep.*,) suggests in *Stomatitis Materna*, that it is not in *every* case dependent on impoverished blood, but may be the result of temporary indigestion ; or of an irritation of the stomach produced by some article of food disagreeing with that organ, or else the effect of some medicinal substance taken purposely or by accident. If constitutional in cause, of course local remedies will fail. He observes:

"There should be a *nourishing diet* immediately prescribed, consisting of plain and simply cooked food which has not been robbed of any of its elements of nutrition. At the risk of being tedious, I would name unbolted wheat meal, lean beef and mutton, garden roots, fruits, etc. ; also light native wines, and lager beer. I would prohibit fine flour, pork, salted meats, smoked fish, corn starch, pastry and confections, excepting simple fruit jellies, unspiced. There should be moderate exercise, fresh air, sunlight in abundance, daily tepid baths, and any other common sense hygienic measures that may suggest themselves.

Medicinal treatment may consist in the exhibition of small doses of mercury, quinine, rhubarb, nitiric, muriatic, and sulphuric acids, or of citric acid. Arsenic also in small doses is very beneficial. Only *one* medicine at a time should be given, and it should be continued at least a week without change, if we would observe its effects. I think the acids will be found most beneficial where there have been mal-nutrition and starvation ; mercury, quinine, and rhubarb, most useful in recent cases of gastric irritation ; and arsenic in cases of great debility and prostration, especially when accompanied by catarrh."

Prof. Alfred C. Post urges a return to the old practice of securing immediate and fixed coaptation of *fractured bones.* Adjustment may require anaesthesia but should be accomplished as speedily as possible, and if needed, extension and counter-extension should be kept up. Permanent mechanical support must be afforded. This, however, necessitates great caution in the use of bandages. The attendants should always be instructed to loosen the bandages at once should painful swelling occur. It is better to leave the case to nature than to endanger irreparable mischief from too tight swathing. Proper support adds greatly to the comfort of the patient, relieving him from his painful sense of helplessness; preventing in a great degree, the spasmodic twitchings which often occasion acute suffering, and diminishing the amount of inflammation consequent upon the injury. We have no doubt that with the proper precautions against excessive pressure from apparatus and appendages, this is really the best practice.

Dr. Chas. Young, of Bellevue Hospital, reports a case of *Rheumatism of the Masseter Muscles Simulating Tetanus.* Robert A., æt. 26 years, single, a native of Ireland, and a weaver by occupation, was admitted to hospital January 6th, with the following history: He had been a healthy man as a general thing, and had never had syphilis; though he confessed to habits of intemperance. Five months before admission to hospital, he had suffered from a sharp attack of acute articular rheumatism in the lower extremities.

Eight days before admission, he had another attack, which began in the joints of the fingers and invaded successively the wrist, elbows, shoulders and back. When seen by the physician, the disease seemed to have settled chiefly in the muscles of the neck, and in both masseter muscles. The latter were so much involved, that the patient was unable to open his mouth sufficiently to receive solid food; and so prominent was this symptom, that, with full knowledge of the co-existence of rheumatism, there were suspicions of tetanus, particular inquiries being made as to previous accidents. The head was thrown slightly backward, and the patient was unable to flex it upon the chest. So firmly was the head maintained in this position, that the patient frequently raised himself in bed by resting upon his heels and head as in a case of opisthotonos.

An anodyne lotion was applied to the affected joints and the parts enveloped in cotton. The patient was placed upon the use of *Potassæ bicarb.* gr. xv, and tr. *Aconiti* ℳ.iij, three times every day. The improvement has been most marked, and there remains only an inconsiderable rigidity of the left masseter muscle.

Professor Pajot, of Paris, proposes the following method of fixing the head in certain cases of *Embryotomy:* "I perforate the cranium in the usual manner, and introduce into the opening a little stick four or five centimeters long and as big as the little finger, to the middle of which a string has already been fastened. I introduce this stick endwise within the cranium by means of a tampooning forceps. When it is completely introduced, I draw upon the string: the stick becomes horizontal, either before or behind, on one side or the other of the pelvis, according as I judge to be desirable. I have then the head entirely at my control. Entrusting the string to an assistant, I apply the instrument which I have chosen, cephalotribe or forceps, without being embarrassed by the string, which takes up no room in the vagina, and I have thus remedied the mobility of the head, a cause giving great difficulty in certain operations, and especially I have remedied it without a new instrument. This, in a word, is my procedure."

Prof. Alvarenga, of Lisbon, describes a variety of *Displacement of the Heart*, involving rotation of that organ. In this case, the base is turned to the right, the apex to the left, the anterior face is above, the right border and part of posterior face look forward, the rest of this face resting upon the diaphragm; the left border looks backward.

Dr. Alex. Bogs commends as a substitute for *Tinct. iodine*, or *Comp. tr. iodine*, the following formula, which is colorless, and will not stain the skin or linen:

℞.—Tr. iod co., - - - - - ℳ48.

Liq. acid carbon c. pur., - - ℳ6.

Glycerine, - - - - - - ℥j.

Aq. destill., - - - - - - ℥v. M.

Dr. Bogs remarks of this preparation—the new product formed, the (*the Phenate, or carbolate of iodine,*) is not only one of the most powerful antiseptics and disinfectants that we possess, but it is likewise a much more curative agent than when the *Solution of iodine* is used alone. I have employed this preparation in all forms, injections, gargles, and lotions, and in all cases in which iodine is indicated. In affections of the throat, ozœna, otorrhœ, and chronic inflammations of the urethra, this medicament constitutes a sovereign remedy ; for, besides its disinfecting property, it modifies the mucous membrane, causes its sensitiveness to disappear, and rids the patient of his disease in less time than when either of these two substances are used separately. It acts in the same manner in leucorrhœa and in chronic blenorrhagia.

An acquaintance of our own, (although not personally a patient), long harrassed by *Dyspepsia*, supposes himself radically cured by the use of the following prescription. The indications it is adapted to fill are sufficiently obvious :

℞.—Pulv. carb. ligni, - - - - ʒij.

Pulv. rad. rhei, opt.,

Sodæ bicarb., - - - - *aa* ʒss.

Pepsin, - - - - - - ℈j.

Eleosach's myristicæ, - - - ℥ss.

Eleosach's menth. pip., - - - ʒij.

M. To be given in divided doses p. r. n. after each meal. Much of the efficiency of the prescription is unquestionably due to the fact that it was made in Paris.

We find the formula for "Shoolbred's Powder," a noted East Indian remedy for *Enlarged Spleen*, in an article by Dr. Joseph Bates, (*Jour. of Mat. Med.*):

℞.—Pulv. jalapae, P. rhei, P. Colombæ, Potassæ bitart., - - - - *aa* ʒj.

Ferri sulphat., - - - - - ʒss.

M. Dose sufficient to open the bowels, three or four times daily.

A very excellent alterative and tonic is afforded in TILDEN'S beautiful *Elixir of iodide of calcium and protoxide of iron.* We have always, previously, been disappointed in securing desired results from the use of the "*Iodide of lime,*" but find this particular preparation to "fill the bill" to our very great satisfaction.

A saturated solution of *Tannin in elixir vitriol* is commended as a prompt and powerful *hæmostatic.* It is said to be useful in both external and internal hæmorrhages.

*Capsicum* having been strongly urged in the treatment of *delirium tremens*, it is stated, as a practical obstacle, that patients, after the first dose, refuse to take it. A correspondent of the *Bost. Med. Jour.* overcomes this difficulty by mixing the *Capsicum* with *Glycerine*, and enclosing it in a gelatine capsule.

The same journal translates and abridges from the *Union Medicale* an account of an operation for *Removal of the Spleen, with Recovery*, by Dr. Pean. The patient was a girl of 20, and the tumor prior to the operation supposed to spring from the left ovary. The operation, under chloroform, lasted about two hours, hæmorrhage being controlled by metallic ligatures and actual cautery. The recovery was singularly rapid and complete. There was no doubt as to the character of the organ removed — which, if our memory serves us, was the case when somebody removed the entire liver from a Cincinnatian, a number of years since, with no perceptible injury either to the subject or the operator.

*Apropos* to surgical enterprises upon the abdominal viscera, we find in the *Medical Record* the report of a case of *Extirpation of the Uterus*, by G. P. HACKENBERG, M.D., of Hudson, N. Y. The operation was undertaken under the diagnosis of an ovarian tumor, and on discovery of its real nature it was too late to recede. The patient died, on the morning of the third day, of Peritonitis. This case foots up with those previously reported — FORTY-EIGHT with EIGHT recoveries. Comment is unnecessary.

Dr. Hackenberg, in closing the report of his case, adds the following suggestions in diagnosis:

In examining further into some of the symptoms of this remarkable case, it appears to me now that we can trace in a measure their true character.

That the menstrual function was not impaired was quite significant, if not pathognomonic in the case. Ovaries being free of disease, with good systemic blood, usually perform their function uninterruptedly; but where one or both are diseased they show menstrual trouble. Even where but one is affected, the other, through sympathy, refuses to perform its office; and their disease always manifests manifold sympathetic troubles. Uterine degeneration, partly under revulsive influences, but more owing to a deterioration of the blood, will likewise suspend the healthy action of the ovaries; but no ex-uterine growth will have the same effect, as long as the blood is left with its normal standard of fibre. Therefore the insidious manner in which the disease developed itself, and the little disturbance it caused to the general system, even after the tumor had grown to a considerable size, should have excited our suspicion, that the disease was neither ovarian nor a malignant degeneration of the uterus. In either case the symptoms would have been more active.

The tumor was movable, or rather floating, as uterine tumors usually are. In Dr. Krackowizer's case the mobility of the tumor was in such a degree, that in the examination of the case there was no agreement from which side it originated. This feature alone should awaken suspicion that the tumor was of central origin.

As a means to make out our diagnosis in this disease, the use of the uterine sound is not to be neglected; but without corroborating symptoms to sustain us, what in our hands it might reveal should be received with a cautious consideration.

The examination, per rectum, is of the greatest importance, as the relative position of morbid growth, within the base of the pelvic cavity, with the uterine organs can be accurately ascertained.

In uterine tumors, the developing symptoms are peculiar. They are the play of physiology more than pathology; being somewhat akin to those of gestation. They manifest themselves not only by characteristic sensations, but leave more or less a print on the os tincæ, the mammary gland, the stomach and the urinary secretions. In uterine tumor the mammary glands are well developed; in ovarian tumor the tendency is to atrophy of these organs.

In uterine tumor there is not the tendency of the body to waste, and the development of cachexia that there is in ovarian tumors and malignant degeneration of the uterus. Some time after the first stages of the disease have passed over, for a period of several months, there is a tendency to obesity. This was the case with my patient.

In the advanced stage of the disease, the pain is likewise characteristic. It is like bearing a heavy weight, paroxysmally manifesting itself in catching, quickening sensations. It is like an ill-defined quickening sensation, common in gestation. The pain in the back is likewise more troublesome in this disease than in ovarian tumor.

In uterine tumor the uterus is more or less in an unusually developed state, with its sensibility exalted. Therefore in menstruation there is more or less either dysmenorrhœa or menorrhagia, if not both. This is not owing to any morbid condition of the ovaries, but simply to the increased sensibility of the uterus, and may be so slight as even to pass the special notice of the patient, her mind being so intently occupied with the main disease. The developed condition of the uterus can but be detected by way of the rectum.

Although dyspnœa may be considerable in all excessive growths of abdominal tumors, in ovarian tumors this symptom is particularly troublesome. There is even a marked difference in this symptom between fibrous and serous tumors of the same kind, the latter causing more difficult respiration than the former. These differences are owing to the more rapid enlargement taking place in the liquid tumors than those that are solid. And paroxysmal dyspnœa may be accounted for, by the sudden changes in the pressure on the diaphragm, caused either by gaseous or fœcal accumulations within certain portions of the alimentary canal.

Mr. H. C. Scorby read a paper lately before the Sheffield Literary and Philosophical Society, in which he set forth the difficulties which the toxicologist encounters in his efforts to prove a case of poisoning by *Belladonna*. These difficulties are obviated by the use of the micro-spectroscope. The spectrum of the juice of *Belladonna* is very distinct, especially when the coloring matter has been added to a solution of *Carbonate of soda.* A small fraction of a single berry is sufficient to produce the spectrum bands characteristic of the *Belladonna.*

Dr. B. W. Richardson (*Medical Times and Gazette*) closes an article on the "*Influence of Extreme Cold on the Nervous Functions*," with the following *resumè:*

1. Nerve tissue in living animals undergoes the process of freezing at 16° Fahr.

2. In the process of freezing any nerve structure the cold acts primarily on the vascular system of the structure, causing, firstly, a stage of preaction, or exaltation of action; secondly, a stage of inertia, or temporary death; and thirdly, in recovery, a stage of reaction, also with an exaltation of action.

3. Nerve substance, when so reduced by cold as to be incapable of conveying sensation, may yet convey an electrical current; but when nerve structure is absolutely frozen through in every part, it ceases entirely to act as an electric conductor.

4. The cerebrum of a living animal may be frozen. In this state the consciousness of the animal is lost, but the functions of organic life remain the same. The animal thus placed is in a state of artificial hybernation from which it may recover. On recovery the brain does not seem to have lost any power. The phenomena are those simply of awaking from profound sleep.

5. The cerebellum may be frozen, and afterward complete restoration of its functions may be secured. In birds, freezing the cerebellum produces backward movements; in rabbits, convulsive muscular action.

6. Complete freezing of the medulla kills by destroying the respiratory power.

7. The spinal column may be frozen and restored. In birds the process of freezing the cervical part of the column produces backward movements with intervals of stupor.

8. By destroying, under the influence of cold, one part of the nervous system, we may pervert the function of another part. Thus, by arresting the functional activity of the cerebral mass, we can exalt the function of the spinal cord; and by destroying the function of the cerebellum, we can exalt the function of the anterior cerebral ganglia, or *vice versa.*

Several cases of *Fatty Degeneration*, which have recently fallen under our notice, lend interest to the following epitome of the clinical characteristics of fatty liver and fatty heart, which we scissor from the *London Lancet:*

1. The enlargement may be considerable, but is rarely so great as that often attained by the waxy liver. It is not often that the anterior or lower border reaches down beyond the umbilicus, or even so far. Occasionally, however, the vertical hepatic dullness is increased out of proportion to the actual amount of enlargement, in consequence of the organ being so soft and flabby that it folds upon itself, so that the anterior margin is depressed, and a larger portion of the organ is brought into apposition with the abdominal parietes.

2. As in waxy disease, the enlargement is tolerably uniform in every direction and there are no circumscribed bulgings, so that the natural form of the liver is but little altered. There is no expansion or bulging of the lower ribs.

3. The enlarged liver is less resistant to pressure and of softer consistence than the waxy disease Owing to its flabbiness it is easily pushed aside by the finger, and when the abdominal parietes are thin, its soft, doughy consistence may be readily appreciated.

4. The outer surface is smooth and the lower margin rounded. Although fatty degeneration may co-exist with cirrhosis, the liver under such circumstances is reduced in size. A lobulated enlarged fatty liver is rarely, if ever, met with.

5. There is no ascites or enlargement of the superficial veins of the abdomen. A large accumulation of oil in the liver so far interferes with the circulation as to lead to an anæmic condition of the liver itself, but not to such an extent as to cause ascites.

6. Even in extreme cases bile continues to be secreted, and its excretion is not arrested or impeded. Jaundice, therefore, is not a symptom of the fatty liver.

7. The same remark applies to pain. Fatty enlargement of the liver from first to last. The organ can be freely manipulated with impunity, although in extreme cases the patient may complain of a feeling of weight or distension in the abdomen, increased by turning on the left side.

8. From the absence of symptoms, few opportunities are afforded of watching the growth of fatty enlargement of the liver, but this is always slow and imperceptible.

9. The constitutional symptoms of fatty liver are few and not characteristic, and those which have been noted are often due, for the most part, to coëxisting fatty degeneration of other organs, and more especially of the heart. General debility and want of tone in the nervous and vascular systems are amongst the most prominent symptoms. The patient is easily tired, and bears depletion of the inroads of acute disease badly. The late Dr. Addison described a condition of the integuments, which he believed to be pathognomonic of fatty degeneration of the liver. "To the eye," he says, "the skin presents a bloodless, almost semi-transparent and waxy appearance. When this is associated with mere pallor it is not very unlike fine polished ivory, but when combined with a more sallow tinge, as is now and then the case, it more resembles a common wax model. To the touch, the general integuments, for the most part, feel smooth, loose, and often flabby; whilst in some well-marked cases all its natural asperities would appear to be obliterated, and it becomes so exquisitely smooth and soft as to convey a sensation resembling that experienced on handling a piece of the softest satin." These appearances are chiefly met with in females, and although they are far from being invariably present, yet in most cases the countenance and general integuments are more or less pasty and anæmic, and sometimes the skin appears greasy from increased action of the sebaceous follicles. Patients with fatty liver also suffer often from dyspeptic symptoms, such as flatulence, hypochondriasis, irregular action of the bowels—usually constipation, but not unfrequently profuse diarrhœa from slight causes.

10. Enlargement of the spleen is rarely present. The portal circulation is not obstructed to such an extent as to lead to enlargement of this organ from stasis of blood; and the spleen is not liable, as in waxy disease, to a deposit of the same material as that which causes the liver to enlarge.

There are, however, certain other organs which are apt to undergo fatty degeneration as well as the liver, and the disease in each of these organs has symptoms of its own, which, when present, will throw light on the nature of the hepatic enlargement. Thus—

11. When there is fatty degeneration of the heart, in addition to the signs already enumerated, there are often—

*a.* A very feeble, or even inappreciable, cardiac impulse.

*b.* Very faint, or even inaudible, cardiac sounds.

*c.* A very slow, or a quick, feeble, and irregular radial pulse.

*d.* Attacks of vertigo, syncope, or pseudo-apoplexy.

*e.* Dyspnœa on slight exertion, and a feeling of sinking at the epigastrium.

12. When there is fatty degeneration of the kidneys, in addition to the signs already enumerated, there will usually be—

*a.* Urine below the normal standard in quantity, oftener turbid than clear, containing much albumen, and depositing numerous oil-casts.

*b.* A tendency to general anasarca.

*c.* Extreme pallor and pastiness of countenance.

13. As in waxy disease of the liver, the diagnosis will often be materially aided by attending to the circumstances under which the enlargement occurs. Many different conditions of the system may give rise to fatty enlargement of the liver, but most of them may be referred to one of the following heads:

*a.* Large accumulation of fat beneath the skin and throughout the body, in persons who for the most part are large feeders and lead indolent lives. It is in this condition that the heart is most likely to participate in the fatty change, and that you will expect to discover the symptoms of fatty heart already referred to. It is patients in this state who are most prone to die of rupture of the heart. In the "Pathological Transactions" you will find several cases recorded in which patients died of rupture of the heart, and where not only was the heart found in a state of fatty degeneration, but the liver was enormously enlarged from fatty deposit, and there was a large accumulation of fat throughout the body.

*b. Alcoholism.*—Persons who drink immoderately of ardent spirits, particularly if they take little exercise, are very subject to fatty liver. Of thirteen persons who died of delirium tremens, Frerichs found the liver very fatty in six. Of two fatal cases of delirium tremens in which an autopsy was made by me in this hospital some years ago, there was considerable fatty enlargement of the liver in both: in one the organ weighed eighty-three ounces; in the other ninety-six ounces. It is under these circumstances that the kidneys often participate in the fatty degeneration.

*c. Phthisis.*—The great frequency of fatty enlargement of the liver in persons suffering from pulmonary consumption has been already referred to under the head of waxy liver.

In consumptive females it is much more common than in males. In this disease, it is not a little remarkable that, while fat disappears rapidly from almost every tissue in the body, it should accumulate in such large quantities in the liver.

*d.* Other wasting diseases besides phthisis—such, for instance, as cancer, simple ulcer of the stomach, and chronic dysentery—are likewise often attended by fatty enlargement of the liver.

It appears, then, that fatty liver is met with under two opposite conditions: one, in which there is an increased supply of material capable of being converted into oil, and where fat often accumulates in all the tissues of the body; the other, in which there is a rapid absorption of fat from all the tissues, with consequent emaciation. Its mode of production in the former case is sufficiently obvious; in the latter, the blood becomes loaded with oily matters derived from the patient's own tissues, and this oily matter is separated from the blood in its passage through the liver. The impaired absorption of oxygen in phthisis, interfering with the proper metamorphosis of the oil, accounts for fatty liver being more common in pulmonary than in other wasting diseases; and the greater frequency of fatty liver in women may be accounted for by women having in general a larger quantity of fat to be absorbed.

The *Journal of Pharmacy* commends as a varnish to exclude the air from excoriated surfaces, sore nipples, burns, erysipelas, etc., a mixture of four parts, by weight, of *yolk of egg* and *Glycerine* triturated thoroughly together. It makes a compound about the thickness of honey, and is unchangeable in the air. It is easily removed by water. Its entirely innoxious properties will often render it eligible.

The *Oxalate of Iron* is commended as a valuable chalybeate by Prof. Schaeffer, of Washington. It is directed in doses of two or three grains, three times a day. It is a protosalt, cheaply made and likely to be pure—is devoid of astringency, although by prolonged use it may have something of that effect. In large doses it will stimulate the circulation to the extent of producing itching over the whole surface of the body. It is, at least, a beautiful looking remedy from its rich color.

Prof. Wm. Pirrie, of the University of Aberdeen, commends in the *London Lancet*, the use of *Carbolic acid* in Burns. Two folds of surgeon's lint, dipped in a liniment of one part of *Carbolic acid* to six parts of olive oil, are closely applied to the whole scalded surface; a double layer of tin foil is placed above the lint, and the whole is secured by a bandage. The air is thus thoroughly excluded, and in ten minutes the patient is free from pain. The bullæ rapidly wither away; suppuration is prevented, and the part is rapidly restored to a healthy condition. The same treatment is applicable after the use of a blister, which proves too severe in its subsequent local influence.

W. M. Coates, Surgeon to the Salisbury Infirmary, reports to the *Medical Times and Gazette* several cases of cure of Enlarged Cervical and other Glands, by injections into their substance of solutions of *Iodine* and *Iodide of potassium*. As much as a drachm of the compound tincture may be used at once. Suppuration is not induced nor scars left behind. Following out the suggestions of the late Prof. Brainard, he has also employed the same process, with success, in Spina Bifida, Ranula, Glandular Tumors of the Mamma with Cysts, Enlarged Bursae, Thick Naevi, Strumous Diseases of the Joints, Encysted Tumors, Bronchoceles, both solid and cystic, etc., etc. Subsequent swelling and pain are to be relieved by poulticing and local or general anodynes.

W. B. Harvey, M.D., of Canton, Miss., (*N. O. Jour. of Med.*) asserts with confidence that Cholera Morbus may be cured, with as much certainty, and within half the time without, as by the employment of opiates. He applies a sinapism to the epigastrium, and administers at once 15 grs. of *Calomel*, floated on a spoonful of water, and allows no more water for two hours. "Speedy relief has been the invariable result,"

which, if true, shows the difference between the climate of Mississippi and Minnesota, for instance. But who, after this, will be prepared to say, *Nulla vestigia retrorsum?*

Prof. Melbourne, in far away Australia, investigating the cause of death from *Snake Bites*, and particularly from that of the cobra de capello, found the blood in all cases very dark, fluid, and incoagulable on exposure to the air. It contained, as estimated, "millions upon millions" of foreign cells, evidently produced at the expense of the blood from the germinal matter of the poison. The blood is thus robbed of its oxygen, with gradual decrease and ultimate extinction of combustion and chemical change in every part of the body, followed by coldness, sleepiness, insensibility, slow breathing, and death. These cells he describes as circular with an average diameter of $\frac{1}{1700}$ of an inch, and containing a nearly round nucleus of $\frac{1}{2800}$ of an inch in breadth, which again contains other more minute spherules of living germinal matter. Other peculiarities distinguish it from the white pus or lymph corpuscle. He considers this a possible clue to the further study of zymotic diseases, cholera, etc., wherein he has been anticipated by many American observers.

Prof. Green, of Maine, (B. M. J.) reports a case of *Fibroid Uterus*, producing great pain by intrapelvic pressure, which caused the patient to demand an operation, whatever the result. Exploratory *abdominal section* was thereupon made, extensive adhesions found, which were believed to preclude attempt at removal of the diseased mass; the wound was closed in the usual manner, and, singularly enough, from the time of the operation, (September, 1866,) has had no return of pain. The uterus has since undergone but little change.

